# Effects of Peanut Shell Flavonoids on Composite Film Properties and Cherry Tomato Preservation

**DOI:** 10.1002/fsn3.4622

**Published:** 2024-12-01

**Authors:** Yuying Li, Kexin Jiang, Qian Mao, Qi Zhao

**Affiliations:** ^1^ Department of Food Science and Engineering, College of Light Industry Liaoning University Shenyang China; ^2^ Department of Information, College of Medicine and Biological Information Engineering Northeastern University Shenyang China

**Keywords:** antioxidant capacity, cherry tomato preservation, composite film, peanut meal alcohol extract, peanut shell flavonoids

## Abstract

This study investigated the effects of varying concentrations of peanut shell flavonoids (PSFs) on the properties of peanut meal extract‐tilapia skin protein composite films and their impact on cherry tomatoes preservation. Peanut meal alcohol extract (Pe) and tilapia skin protein (Co) were used as base materials, combined with PSFs to prepare composite films with excellent antioxidant properties. The results demonstrated that the optimized composite films exhibited superior mechanical properties, with a tensile strength of 9.83 MPa and an elongation at break of 204.04%. Increased flavonoid content enhanced the films' antioxidant capacity, achieving superoxide anion and DPPH radical scavenging rates of 15.08% and 21.37%, respectively. The microstructure, IR spectra, and circular dichroism spectra of the composite films are also significantly different with the change of flavonoid content. In the cherry tomato preservation experiment, the PeCo‐0.5 composite film treatment group maintained a low weight loss rate (11.18%) and malondialdehyde content (13.33 μmol/g) after 15 days, delayed the peak respiration rate, and significantly reduced the peak respiration intensity (3.43 mgCO_2_∙ mg^−1^∙gh^−1^). At the same time, the activity of polyphenol oxidase in the tissue of Cherry Tomatoes was significantly inhibited, and the decrease rate of Vc content was also significantly decreased, which effectively preserving the bright color, smooth appearance, and good aroma of the cherry tomatoes. This study not only provides new insights into the comprehensive utilization of tilapia skin and peanut by‐products but also opens new avenues for the development and application of fruit and vegetable preservation films.

## Graphical abstract

This study demonstrates that peanut shell flavonoids can effectively enhance the mechanical properties of composite films and significantly extend the shelf life of cherry tomatoes. Incorporating peanut shell flavonoids into the films notably improves their antioxidant capacity, with the PeCo‐0.5 film achieving a DPPH radical scavenging rate of 21.37%. In preservation experiments with cherry tomatoes, the PeCo‐0.5 film effectively reduced weight loss to 11.18% and limited malondialdehyde content to 13.33 μmol/g after 15 days, while also delaying the respiration peaks.

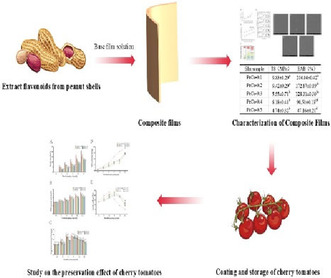

## 1 Introduction

After cherry tomatoes are harvested, although the nutritional supply ceases, metabolic activity within the fruit continues. Shortly after harvested, the fruit experiences a respiratory surge, leading to its transition from a fresh state to senescence (Boe 1971). Additionally, the cellular tissue within the fruit is highly susceptible to contamination by external pathogenic microorganisms, triggering complex biochemical reactions that further accelerate the aging process (De Souza et al. 2015). Pectin, a soluble pectinic acid, is produced within the fruit and is subsequently hydrolyzed into sugar components, leading to a reduction in tissue firmness and adversely affecting the texture of the flesh (Takizawa et al. 2014). Concurrently, enzyme activity changes disrupt the fruit's physiological balance, further accelerating the aging process. A marked increase in malondialdehyde (MDA) levels, resulting from membrane lipid peroxidation, exacerbates aging and reduces the overall quality of the fruit (Zaouali et al. 2020).

Since the advent of plastic packaging, it has greatly enhanced convenience in daily life. However, as the use of plastic packaging has become increasingly widespread, the resulting environmental issues have grown more severe. Consequently, there has been a growing interest in the development of edible packaging materials that are both biodegradable and safe. Despite their potential, edible composite films made primarily from polysaccharide or protein face certain limitations, including high water solubility and insufficient antimicrobial properties. To enhance the preservation performance of these films, natural active ingredients such as flavonoids (Li, Guo, et al. 2024), tea polyphenols (Li, Hua, et al. 2024), and curcumin (Li, Zhang, et al. 2024) have been incorporated into film formulation. Compared to other packaging materials, these enhanced composite films exhibit excellent antioxidant, barrier, and mechanical properties, making them highly suitable for the preservation of fruits and vegetables (Du et al. 2023; Li, Li, et al. 2024; Lv et al. 2024; Shi, Liu, and Wang 2024).

A variety of by‐products are generated during peanut processing, including peanut meal and peanut shells. Peanut meal, the primary by‐product of peanut oil extraction, is rich in protein, sugar, flavonoids, and other nutrients, with protein content comprising approximately 40% of its total mass (Zhong et al. 2017). We developed an active film using modified peanut meal protein isolate (PPI) combined with thymol (TML). The film demonstrated a high total phenol content and exhibited substantial antioxidant and antibacterial activity, indicating that the modified PPI film has considerable preservation potential. Additionally, this film provides a novel approach for creating active food packaging that utilizes PPI. Active components such as polyphenols in peanut meal are typically more hydrophilic than lipophilic, making extraction with organic solvents like ethanol, or their combination with water, more effective in enhancing extraction efficiency and product purity (Mohamed et al. 2024). Peanut polysaccharide is the second most abundant component in peanut meal, with total soluble sugars accounting for 32.5% of its total weight (Ye et al. 2021). Studies have confirmed that peanut polysaccharides possess both antibacterial and antioxidant (Jiang, Ma, and Yan 2014) properties. The active polyphenol content in mature peanut shells can reach 18.11 ± 0.88 mg GAE/g (Liu, Wang, and Zhang 2022). Flavonoids in peanut shells exhibit a range of physiological activities and possess strong antioxidant properties, making them effective natural antioxidant. These characteristics enable flavonoids to extend the shelf life of foods, thereby promoting their widespread application in the food and health products industry. Wang et al. (2024) prepared a composite film using sodium alginate and carrageenan, incorporating peanut shell flavonoids (PSFs) as the active ingredient. The study characterized the physicochemical properties of the PSFs‐SAC composite films and evaluated their effectiveness in preserving chilled pork stored at 4°C. Results indicated that the incorporation of PSFs significantly enhanced the mechanical properties, barrier properties, thermal stability, and antioxidant capabilities of the SAC composite films (*p* < 0.05). Furthermore, the PSFs‐SAC composite films successfully extended the shelf life of chilled pork from 6 days to 12 days.

The collagen content in fish skin can exceed 30% of its total mass, making it an excellent raw material for protein production. Additionally, marine collagen has been widely utilized in various fields, including film preparation and food preservation, due to its superior film‐forming properties, high safety, and broad acceptance (Barzkar et al. 2023). Therefore, extraction protein from waste fish skin not only enhances the value of fish processing by‐products but also promotes their high‐value utilization. Tilapia ranks second in global aquaculture production (Yan et al. 2020). Compared to other fish, tilapia offers the advantages of low cost and abundant resources, making tilapia skin an increasingly popular raw material for protein film preparation.

The aim of this study was to develop an edible composite film using an alcohol extract of peanut meal (Pe) and tilapia fish skin protein (Co), with PSFs as a modifier, and to evaluate its effectiveness in preserving cherry tomatoes. This study will characterize the mechanical properties, antioxidant activity, and fundamental structure of the PeCo composite film, and investigate the effects of varying concentrations of PSFs on the physicochemical properties of cherry tomatoes. The goal is to develop a novel composite film for fruits and vegetables, offering a new avenue for the utilization of peanut and fish processing by‐products.

## 2 Materials and Methods

### 2.1 Materials

Peanut meal ethanol extract: protein content 39%, polysaccharide content 23%, polyphenol content 0.05%, laboratory‐prepared; Tilapia fish skin protein: crude protein content 82.62%, collagen content approximately 53.23%, fat content 3.16%, moisture content 9.78%, ash content 1.78%, laboratory‐prepared.

Glycerin (purity ≥ 99.5%) and ethanol (purity ≥ 95.0%) were purchased from Tianjin Yongda Chemical Reagent Co. Ltd. (Tianjin, China). 1,1‐Diphenyl‐2‐pyridinehydrazyl (DPPH) radical (purity ≥ 98.0%) was obtained from Jiangsu Vorradex Company.

### 2.2 Preparation of Peanut Shell Flavonoids

The extraction of flavonoids from peanut shell was performed following the method of Ma et al. (2013), with slight modifications. The specific procedure is as follows: First, the peanut shell was crushed and pretreated, followed by sieving through an 80‐mesh screen. The sieved material was then subjected to ball milling at 600 rpm for 60 min with anhydrous ethanol. Subsequently, double aqueous leaching was performed. Precisely 1.8 g of peanut shell powder and 9 g of dipotassium hydrogen phosphate were weighted and placed in a beaker, to which 15 mL of ethanol and 12 mL of water were added. The mixture was stirred thoroughly. The beaker containing the mixture was then placed on a magnetic stirrer with the temperature set to 55°C and the stirring speed adjusted 600 rpm, and the leaching was allowed to proceed for 2 h. After extraction, the solution was centrifuged (CR_21_N, Hitachi, Tokyo, Japan) at 574 × g (2000 rpm, R_15_A rotor) at 4°C for 10 min, and the supernatant was collected. The supernatant was decolorized using activated carbon. Finally, the supernatant was subjected to rotary evaporation to obtain a concentrated extract, which was subsequently spray‐dried to yield PSFs.

### 2.3 Preparation of the Films

To prepare the composite film, 1 g of peanut meal alcohol extract and 1 g of tilapia fish skin protein were dissolved in 100 mL of distilled water, resulting in a base film solution with concentrations of both peanut meal alcohol extract and fish skin protein at 1.0%. This solution was designated as the PeCo composite film solution. The PeCo base film solution was then placed in a water bath at 50°C and stirred while heating for 30 min. Peanut shell flavonoids were added to the base film solution in the amounts of 0.1 g, 0.2 g, 0.3 g, 0.4 g, and 0.5 g, creating five composite film solutions with PSF mass concentrations of 0.1%, 0.2%, 0.3%, 0.4%, and 0.5%, respectively. These were designated as PeCo‐0.1, PeCo‐0.2, PeCo‐0.3, PeCo‐0.4, and PeCo‐0.5. Each film solution was homogenized with 0.5% glycerol at 10,000 rpm for 1 min (XHF‐DY, Sandoz Biotechnology Co. LTD., Zhejiang, China), followed by ultrasonic treatment for 10 min to eliminate any bubbles. The purpose of eliminating bubbles during the preparation of the composite film is to ensure a homogeneous and uniform film structure, which enhances to the quality and functionality of the final composite film. After defoaming, the film solution was poured into plastic plates and dried in a 60°C thermostatic incubator for 21 h. The dried films were then removed and equilibrated in a glass desiccator at a relative humidity of 75% for 24 h before being carefully peeled off to obtain the final composite films.

### 2.4 Characterization of the Films

#### 2.4.1 Mechanical Properties

The mechanical properties of the composite films were evaluated using a texture analyzer (CT3, BROOKFIELD, USA), focusing on elongation at break (EAB) and tensile strength (TS). The films were cut into strips measuring 2 cm × 3 cm and secured with a TA‐GPA model clamp. The test speed was set to 2 mm/s with a load of 2 g. The tensile strength and elongation at break were calculated using the following formulas:

(2.1) $$\mathrm{TS}\;\left(\mathrm{MPa}\right)=\frac{F}{d\times w}$$

where F is the maximum tensile force at fracture (N), d is the thickness of the composite film (mm), and w is the width of the composite film (mm).

(2.2) $$\mathrm{EAB}\;\left(\%\right)=\frac{L_{2}-L_{1}}{L_{1}}\times 100$$

where L_2_ is the maximum tensile length at fracture (mm), and L_1_ is the original length of the composite film (mm).

#### 2.4.2 Water Vapor Permeability (WVP)

With additional refinements, the water vapor permeability of the composite film was assessed using an adapted method from Wu et al. (2013). The composite film was first cut into 6 cm diameter discs. A glass weighing bottle with a diameter of 5 cm was filled with 20 mL of distilled water, and Vaseline was applied to the bottle's mouth. Vaseline is used to create a tight seal over the mouth of the glass weighing bottle during the water vapor permeability (WVP) test. This ensures that the film disc is securely attached, preventing any leakage and enabling an accurate measurement of water vapor transmission through the film. The film disc was then placed over the bottle's mouth, gently pressed to ensure a tight fit, and secured with a rubber band. The total mass (M_0_) of the bottle was recorded, after which it was placed in a glass desiccator containing silica gel to maintain relative humidity at 100% on one side of the film and 0% on the other. The desiccator was then placed in a constant temperature incubator at 25°C. After 8 h, the bottle was reweighed (M₁), and the water vapor permeability of the composite film was calculated using the following formula:

(2.3) $$\mathrm{WVP}\;\left(g\;m^{-1}\;{\mathrm{Pa}}^{-1}\;s^{-1}\right)=\frac{\left(M_{0}-M_{1}\right)\times d}{t\times S\times ∆P}$$

where d is the film thickness (m), t is the test time (s), S is the effective area of the film (m^2^), and ∆P is the water vapor pressure difference between the two sides of the film at 25°C (3168 Pa).

#### 2.4.3 Antioxidant Ability

##### 2.4.3.1 DPPH Free Radical Scavenging Ability

The DPPH free radical scavenging ability of the composite films was evaluated using a slightly modified method from Kaewprachu et al. (2018). Three mL of film samples from each group was placed in test tubes, to which 3 mL of DPPH ethanol solution (0.2 mmol/L) and 3 mL of anhydrous ethanol were added. The control group used anhydrous ethanol in place of the DPPH ethanol solution, while the blank group used distilled water instead of the sample. The test tubes were then kept in the dark for 30 min. After incubation, the absorbance at 517 nm was measured using an ultraviolet spectrophotometer. The sample group was recorded as A_sample_, the control group as A_control_, and the blank group as A_blank_. The DPPH scavenging rate of the composite film sample was calculated using the following formula:

(2.4) $$\begin{array}{c} \text{DPPH free radical scavenging ability}\;\left(\%\right)\\ =\left(1-\frac{A_{\text{sample}}-A_{\text{control}}}{A_{\text{blank}}}\right)\times 100\% \end{array}$$

##### 2.4.3.2 Superoxide Anion Scavenging Capacity

The superoxide anion scavenging capacity of the composite films was measured based on the method by Zhao et al. (2016), with minor adjustments. Nine mL of Tris‐HCI buffer solution (50 mmol/L, pH 8.2) was placed in a test tube, followed by the addition of 8 mL of distilled water and 0.4 mL of film solution of different concentrations. The mixed solution was incubated in water at 25°C for 10 min. Subsequently, 0.6 mL of pyrogallol solution (3 mmol/L) was added, and the absorbance at 320 nm was immediately measured, denoted as A. For the control group, distilled water was used instead of the sample, and the absorbance was recorded as A_0_. The superoxide anion scavenging rate of composite film sample was calculated using the following formula:

(2.5) $$\text{Superoxide anion radical clearance}\;\left(\%\right)=\frac{A_{0}-A}{A}\times 100\%$$

#### 2.4.4 Circular Dichroism (CD) Spectrum

Following the method outlined by Wang et al. (2019), samples with a concentration of 0.2 mg/mL were prepared to analyze the secondary structure changes in peanut meal alcohol extract, fish skin protein, and composite films using a circular dichroism (CD) spectrometer. The experiment was conducted at a temperature of 25°C, with a sample cell path length of 2 mm, and the sensitivity set to 100 mdeg/cm. The CD spectra of the samples were scanned over a wavelength range of 180–260 nm. Each sample was scanned three times to obtain the background, and the average values were used to calculate the protein secondary structure content.

#### 2.4.5 Fourier Transform Infrared Spectroscopy (FTIR)

The method of Arfat et al. (2014) was followed with slight modifications. After drying for 2 h, the composite film was cut into 4 cm × 5 cm strips and mounted on the sample holder. Infrared spectroscopy was conducted after subtracting the air background. The scanning resolution was set to 4 cm^−1^, with a wavenumber range of 4000~500 cm^−1^.

#### 2.4.6 Scanning Electron Microscopy (SEM)

The balanced composite film sample was mounted on the sample holder and coated with a thin layer of gold. Surface morphology of the composite film was observed using a HITACHI S‐4800 scanning electron microscope at an accelerating voltage of 5.0 kV, with a magnification of 2000×.

### 2.5 Study on the Preservation Effect of Cherry Tomatoes

#### 2.5.1 Coating and Storage

The preparation of PeCo film solutions with flavonoid concentrations of 0.1%, 0.2%, 0.3%, 0.4%, and 0.5% followed the same procedure as described in section 2.3. For this experiment, we used “Millennium” cherry tomatoes purchased from the Qiansheng market. Six groups of cherry tomatoes were randomly selected, with the equal number of tomatoes in each group. These groups were designated as the control group, and the CoPe‐0.1, CoPe‐0.2, CoPe‐0.3, CoPe‐0.4, and CoPe‐0.5 treatment groups, respectively. The tomatoes in each treatment group were soaked in their respective solutions for 3 min and then removed and allowed to dry naturally, while the control group received no treatment. All tomatoes were stored in a constant temperature incubator at 4°C for 15 days. Indicators, including weight loss rate, polyphenol oxidase (PPO) content, respiratory intensity, and other relevant parameters, were measured every 3 days, with three fruits selected for parallel experiments.

#### 2.5.2 Weight Loss Rate

Cherry tomatoes are susceptible to mechanical damage during storage and transportation, leading to water and nutrient loss, which in turn reduces fruit weight and market value. To assess this, the initial weight W_0_ of each tomato was recorded, and the weight W was measured every 3 days. The weight loss rate was calculate using the following formula:

(2.6) $$\text{Weightlessness rate}\;\left(\%\right)=\frac{W_{0}-W}{W_{0}}\times 100$$

#### 2.5.3 Polyphenol Oxidase (PPO) Content

To measure the PPO content, 3 g of cherry tomato pulp was weighted and recorded as the sample weight W (g). The pulp was then with 3 mL of sodium acetate buffer (PH5.5) and thoroughly ground. The mixture was centrifuged (CR_21_N, Hitachi, Tokyo, Japan) at 10,000 r/min for 10 min at 4°C, and the volume of the supernatant was recorded as V (mL). Next, 2 mL of catechol solution (0.05 mol/L) and 8 mL of sodium acetate buffer (0.05 mol/L, pH 5.5) were added to a test tube, followed by 0.2 mL of the extraction solution, recorded as V_1_ (mL). The mixture was shaken well, and the absorbance change at 420 nm was measured, recorded as ∆OD_420_. The reaction time t (min) was also recorded. The PPO content was calculated using the following formula:

(2.7) $$\mathrm{PPO}\;\text{content}\;\left(U\right)=\frac{∆{\mathrm{OD}}_{420}\times V}{W\times t\times V_{1}}$$

#### 2.5.4 Respiratory Intensity

Respiratory intensity is determined by measuring the amount of carbon dioxide (CO₂) released by the fruit over a specific period. First, place 20 mL of sodium hydroxide solution (0.5 mol/L) in a Petri dish and position it at the bottom of a desiccator. Weigh the cherry tomatoes and record the mass as m. Place the tomatoes in a beaker, which is then set on a platform inside the desiccator. Seal the desiccator and record the sealing time h. After the designated time, transfer the sodium hydroxide solution to a beaker and rinse the Petri dish several times with distilled water. Five mL of saturated barium chloride solution is added to the beaker, and the mixture is then shaken thoroughly to ensure complete precipitation occurs. Then, add 20 μL of 1% phenolphthalein solution and titrate with oxalic acid solution (0.2 mol/L) until the solution changes color, maintaining the color for at least 30 s. Record the volume of oxalic acid solution consumed as V. The respiration intensity, expressed as the amount of CO₂ produced, can be calculated using the following formula:

(2.8) $$\text{Respiration intensity}\;\left(\mathrm{mg}\;\left(\mathrm{\mu L}\right)/\left(h∙g\right)\right)=\frac{\left(V_{\mathrm{CK}}-V\right)\times C\times M}{m\times h}$$

where V_CK_ is the control titration volume, V is the sample titration volume, C is the concentration of the oxalic acid solution, and M is the molar mass of CO₂.

#### 2.5.5 Malondialdehyde (MDA)

The MDA content was measured following the GB 5009.181‐2016 standard. First, 4 g of peeled cherry tomato pieces was weighted, and the mass recorded as m. The pieces were then homogenized in 10 mL of 10% trichloroacetic acid (TCA) solution, followed by filtration to collect the filtrate, with the total volume recorded as V. Next, 5 mL of the filtrate was mixed with 5 mL of 0.6% thiobarbituric acid (TBA) solution, and the mixture was thoroughly shaken before being heated in a boiling water bath for 20 min. After cooling the reaction mixture under running water, the absorbance was measured at wavelengths of 450 nm, 532 nm, and 600 nm, recorded as A_450_, A_532_, and A_600_, respectively. The MDA content was calculated using the following formula:

(2.9) $$\mathrm{MDA}\;\text{content}\;\left(\mu \;\mathrm{mol}/g\right)=\frac{V}{m}\times \left(6.45\;\left(A_{532}-A_{600}\right)-0.56A_{450}\right)$$

#### 2.5.6 Vitamin C (Vc) Content

The Vitamin C (Vc) content was determined using the 2, 6‐dichloroindophenol method. First, 5 g of peeled cherry tomato pulp was weighed, recorded as M, and then mixed with 5 g of 2% oxalic acid solution. The mixture was thoroughly ground, and 2 g of the ground material was taken and diluted with oxalic acid solution to a total volume of 20 mL. The solution was then filtered to obtain the filtrate. Next, 1 mL of the filtrate was titrated with a 2, 6‐dichloroindophenol solution of known concentration C until a stable color change was observed for at least 30 s. The volume of 2, 6‐dichloroindophenol solution was recorded as V_1_. A blank titration was performed by replacing the filtrate with distilled water, and the corresponding volume was recorded as V_0_. The Vc content was calculated using the following formula, where A is the dilution factor of the sample, and T is the titration factor of the 2, 6‐dichloroindophenol solution (mg/mL), M is the sample quality:

(2.10) $$\mathrm{Vc}\;\text{content}\;\left(\mathrm{mg}/100\;g\right)=\frac{\left(V_{1}-V_{0}\right)\times A\times T}{M}\times 100$$

### 2.6 Statistical Analysis

Statistical analysis of the composite film properties and the preservation parameters of cherry tomatoes was performed using the Duncan's multiple range test in SPSS Statistic 26 (IBM Corporation, New York, USA). The experimental results are presented as the mean ± standard deviation (SD) of three replicates. In the charts, different lowercase letters indicate significant differences within groups, while different uppercase letters denote significant differences between groups (*p* < 0.05).

## 3 Results and Discussion

### 3.1 Characterization of the Composite Films

#### 3.1.1 Mechanical Properties

As shown in Table 1, the tensile strength (TS) of the PeCo composite film decreased with increasing amounts of PSFs. When the flavonoid content was 0.1%, the TS of the PeCo composite film was 9.83 MPa. However, as the flavonoid content increased to 0.5%, the TS reduced to 4.74 MPa, marking a 51.78% decrease. A similar trend was observed in the elongation at break (EAB) of the composite film, which also progressively decreased with the increased flavonoid content. The film containing 0.1% flavonoids exhibited the highest flexibility, with an elongation at break of 204.04%. This reduction in mechanical properties may be attributed to the increasing mass concentration of flavonoids, which likely leads to a rise in the overall molecular weight of the composite film system. Consequently, this promotes aggregation of the internal components, diminishing the stability of the network structure and increasing the proportion of large pores within the film's architecture. These structural changes contribute to the gradual decline in tensile strength (Zheng et al. 2023). Additionally, the internal structure of the composite film undergoes a transition from a compact arrangement to a more loose and porous configuration, which increases the film's brittleness and decreases its flexibility, leading to a progressive reduction in elongation at break.

**TABLE 1:** TS and EAB of film.

Film sample	TS (MPa)	EAB (%)
PeCo‐0.1	9.83 ± 1.29^a^	204.04 ± 0.42^a^
PeCo‐0.2	9.42 ± 0.29^a^	172.87 ± 0.19^ab^
PeCo‐0.3	5.55 ± 0.71^b^	128.31 ± 0.30^bc^
PeCo‐0.4	6.18 ± 0.41^b^	98.51 ± 0.13^cd^
PeCo‐0.5	4.74 ± 0.32^b^	47.16 ± 0.21^d^

*Note:* ^a‐d^different superscripts within the same column indicate significant differences (P < 0.05).

#### 3.1.2 Water Vapor Permeability (WVP)

**FIGURE 1:**
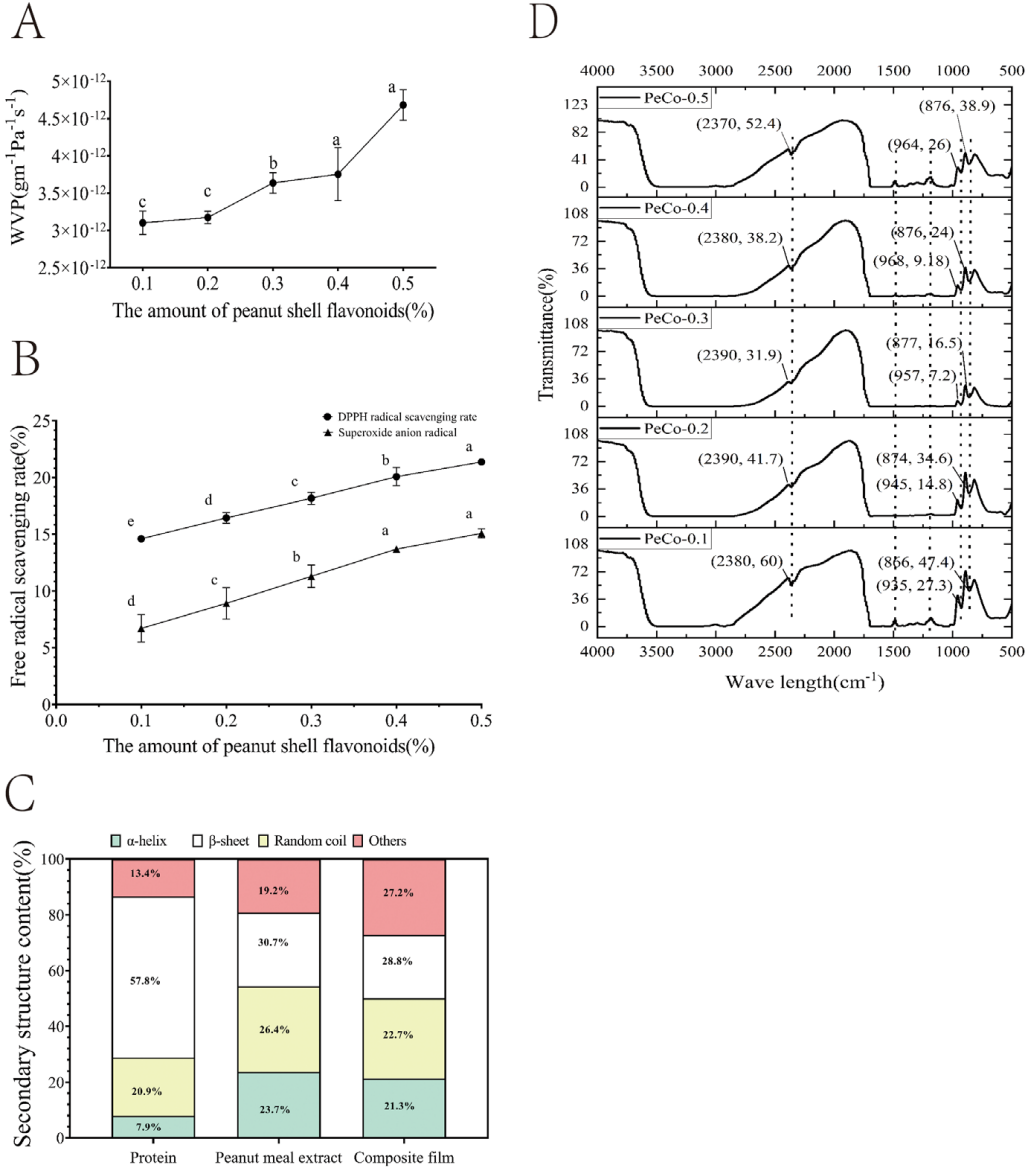
Effects of peanut shell flavonoids on antioxidant capacity and surface microstructure of PeCo films. (A: WVP, B: Antioxidant ability, C: Circular dichroism spectrum, D: Fourier infrared spectroscopy).

Figure 1A illustrates that the water vapor permeability of the composite film increased with higher mass concentrations of PSFs. This increase in WVP may be attributed to the higher flavonoid content, which likely increases the system's molecular weight, resulting in more porous film structure and thus greater water vapor transmission. Since water plays a critical role in the deterioration of food products (Rubilar et al. 2013), the observed increase in water vapor permeability could potentially reduce the effectiveness of the composite film as a preservative.

#### 3.1.3 Antioxidant Ability

According to Figure 1B, the DPPH free radical scavenging rate of the PeCo‐0.1 composite film was 14.60%. When the flavonoid concentration was increased to 0.5%, the scavenging rate rose to 21.37%, which was significantly higher than that of the PeCo‐0.4 composite film group (*p* < 0.05). This indicates that the DPPH free radical scavenging concentration of the composite film improves with increasing flavonoid content. A composite film with strong antioxidant capacity can slow down food spoilage and extend shelf life. This finding aligns with the research of Wang et al. (2024), who prepared sodium alginate‐carrageenan composite films incorporating PSFs as an antioxidant. Their study demonstrated that the antioxidant activity of the composite films significantly improved, with the DPPH and ABTS free radical scavenging rates increasing as the concentration of PSFs was elevated. Specifically, the PSFs‐SAC composite films effectively extended the shelf life of chilled pork from 6 days to12 days by inhibiting microbial growth and lipid oxidation. Additionally, the scavenging rate of superoxide anion radicals also improved with higher flavonoid content. When the flavonoid contents were 0.1%, the superoxide anion radical scavenging rate was 6.71%. Increasing the flavonoid content to 0.5% elevated the scavenging rate to 15.08%, representing a 1.24‐fold increase. However, the overall scavenging rate of superoxide anion radicals was lower than that of DPPH free radical, likely due to the stronger scavenging ability of flavonoids against DPPH radicals. In summary, as the flavonoid content in the composite film increases, its antioxidant capacity is progressively enhanced, thereby improving its effectiveness in preserving food.

#### 3.1.4 Circular Dichroism (CD) Spectrum

Figure 1C presents an analysis of the secondary structure content of proteins in fish skin protein, peanut meal alcohol extract, and the PeCo‐0.5 composite film. Circular dichroism (CD) spectroscopy is a powerful tool for revealing the spatial configuration of proteins, allowing for detailed analysis of their secondary structures. As illustrated in Figure 1C, compared to fish skin proteins, the composite film exhibited an increase in the content of α‐helix and random coil structures, while the β‐folded or sheet structure content significantly decreased by approximately 29%. When compared to the peanut meal alcohol extract, the α‐helix structure in the composite film also increased, whereas the β‐sheet and random coil structures decreased. These observations suggest that the number of hydrogen bonds between the carbonyl oxygen of the peptide bond and the N–H group on the protein peptide chain within the composite film has increased, while the number of hydrogen bonds between N–H and C=O has decreased. This alteration may be attributed to the interaction between the PSFs and proteins in the composite film, leading to changes in the protein's secondary structure. Additionally, polyphenols present in the peanut meal alcohol extract may form complexes with PSFs, thereby increasing the number of covalent bonds, such as C–N or C–S bonds (Zhao, Yu, et al. 2020; Zhao, Huang, et al. 2020). In summary, the addition of PSFs not only influences the physical properties of the composite film but also alters the secondary structure of the proteins within the film through interactions with the proteins.

#### 3.1.5 FTIR

Figure 1D shows the Fourier infrared spectrum of the composite film, highlighting three characteristic absorption peaks within the wavelength ranges of 2370–2390 cm^−1^, 935–964 cm^−1^, and 866–877 cm^−1^. The spectrum exhibits fewer absorption peaks in the 2400–2100 cm^−1^ range, corresponding to the region of triple bond and double bond accumulations. The absorption peak around 964 cm^−1^ is attributed to the out‐of‐plane bending vibration of the methyl group at the terminus of the pyran ring, while the peak at 877 cm^−1^ is associated with the C–H bending vibration of the equatorial bond at the pyran ring's terminus (Dang and Yoksan 2015). Additionally, three flattopped valleys were observed in the wavelength ranges of 2800–3500 cm^−1^, 1500–1700 cm^−1^, and 1000–1250 cm^−1^. These flat‐topped peaks likely result from the excessive absorption intensity of sample molecules within these bands, leading to supersaturation and the formation of flat peaks. Specifically, the flat‐topped peak around 2850~2900 cm^−1^ may be attributed to the dense overlapping of characteristic peaks from the amide A and amide B bands, while the flat‐topped peaks within the 1000~1700 cm^−1^ range may be due to the presence of the amide Ι, amide II, and amide III bands.

#### 3.1.6 SEM

**FIGURE 2:**
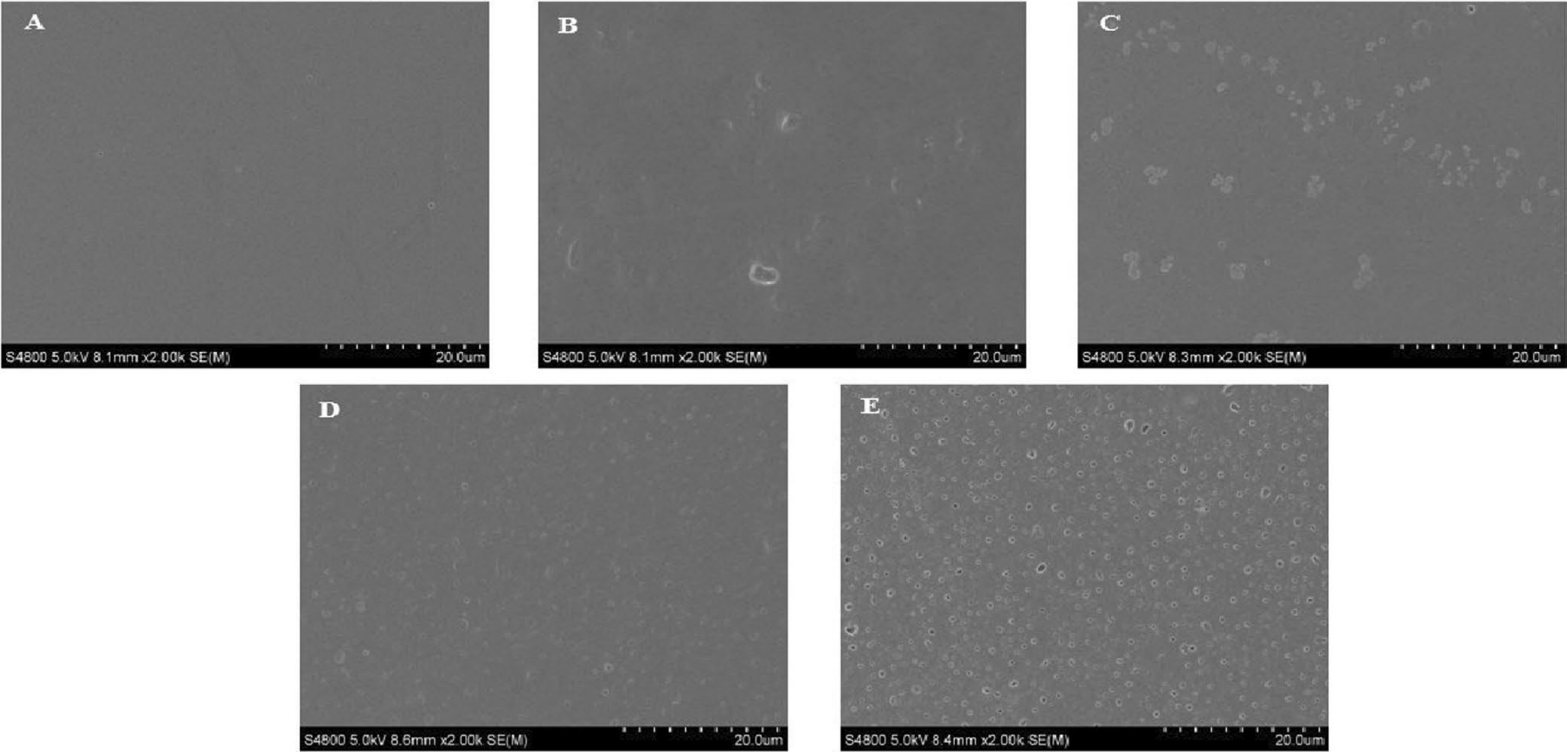
Effect of peanut shell flavonoids on the surface microstructure of PeCo films. (A: PeCo‐0.1, B: PeCo‐0.2, C: PeCo‐0.3, D: PeCo‐0.4, E: PeCo‐0.5).

The surface microstructure of composite films with varying flavonoid content is shown in Figure 2. Scanning electron microscopy (SEM) was employed to examine the surface morphology of the films, including smoothness, cracks, and other microstructural features. These observations provide insights into the interactions within the internal matrix of the composite films and help elucidate the differences in mechanical and barrier properties observed among the different formulations (Pan et al. 2014). The surface of the composite films in each group appeared smooth, uniform, and continuous, with no noticeable depressions or convex particles. This suggests that the flavonoids, peanut meal alcohol extract, and fish skin protein exhibited good compatibility, resulting in a homogeneous dispersion of these components within the film‐forming solution. As shown in the figure, the porosity of the composite films increased progressively with the higher concentrations of PSFs. The PeCo‐0.1 composite film exhibited only a few pores; however, as the flavonoids content increased to 0.5%, a large number of dense pores became apparent. This observation is consistent with the results of the water vapor transmission rate for the composite films. Overall, the variation in flavonoid content did not significantly alter the structural integrity of the composite films, as they remained densely packed with no fractures or large holes, indicating that they can effectively meet preservation requirements (Wang et al. 2013).

### 3.2 Cherry Tomatoes Preservation Effect

#### 3.2.1 Weight Loss Rate

**FIGURE 3:**
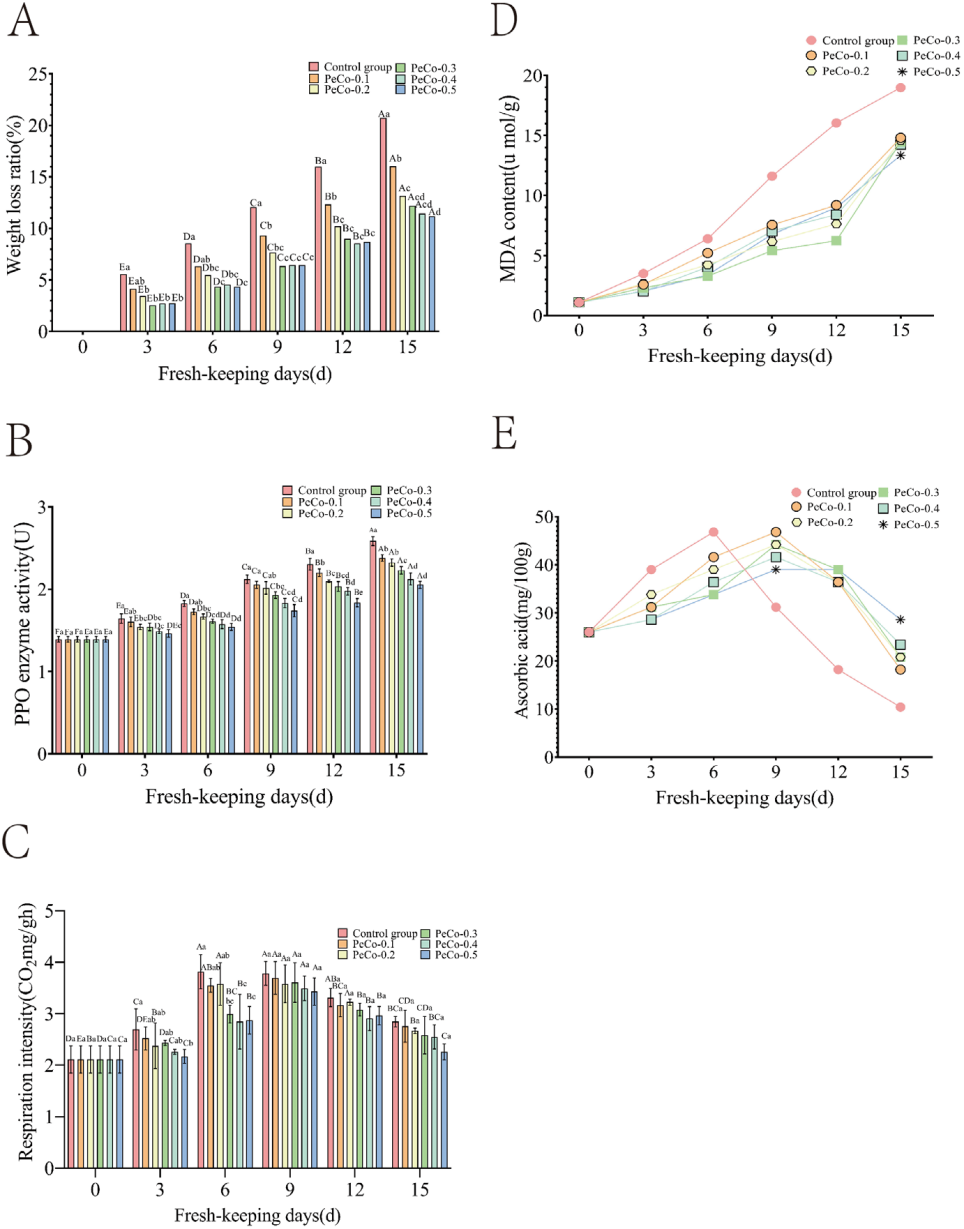
Quality index of cherry tomatoes changed within 15 days. (A: Weightlessness rate, B: PPO content, C: Respiratory intensity, D: MDA, E: Vc content).

The weight of cherry tomatoes was monitored over a 15‐day period, with the results presented in Figure 3A. Both control and treatment groups exhibited an increasing trend in weight loss over the 15 days. This increase is primarily attributed to the gradual decrease in internal water content due to respiration during storage. As carbon atoms in the tissues are released in the form of carbon dioxide, the surface of cherry tomatoes begins to shrink, leading to a reduction in fruit weight (Akhtar et al. 2023). Consequently, the weight loss continued to increase over time. The control group exhibited a significantly higher weight loss rate compared to the treatment groups. By Day 3, the weight loss rate in the treatment groups with flavonoid concentrations exceeding 0.2% was significantly lower than that of the control group (*p* < 0.05), indicating that the composite film effectively inhibited water loss and significantly reduced the weight loss rate of cherry tomatoes during storage. Additionally, the data suggest that when the flavonoid content in the composite film exceeds 0.2%, further increases in flavonoid concentration do not have a significant impact on the fruit's weight loss rate during storage (*p* > 0.05).

#### 3.2.2 PPO Content

As demonstrated in Figure 3B, the PPO enzyme activity in the cherry tomatoes tissue significantly increased with the duration of storage. The PPO activity in control group was significantly higher than that in the treatment groups (*p* < 0.05). For the same storage duration, the PPO enzyme activity in the treatment groups decreased as the flavonoid content in the composite film increased. This trend is likely due to the fact that PPO enzyme activity is highly influenced by O₂ concentration. The perishability of fruits and vegetables is caused by various browning enzymes and cell wall‐degrading enzymes such as PPO and peroxidase (Punia Bangar et al. 2022). The melanin present in the black spots that form on the surface of rotting fruits and vegetables is an indirect product of PPO catalysis (Zhao, Yu, et al. 2020; Zhao, Huang, et al. 2020). PPO catalyzes the oxidation of free phenolic acids in fruits and vegetables, leading to the formation of quinones. These quinone can then condense with proteins in the tissues or with each other to produce dark pigments, ultimately causing browning and deterioration of the produce (Tinello and Lante 2018). The increased flavonoid content in the composite film imparts strong antioxidant properties (Zengin et al. 2023), which can lower the O_2_ concentration within fruit tissues. This reduction in oxygen availability slows down the rate of increase and the peak value of PPO enzyme activity. When the flavonoid content exceeds 0.4%, further increases do not have a significant effect on PPO enzyme activity in the fruit (*p* > 0.05).

#### 3.2.3 Respiratory Intensity

Changes in respiratory intensity of cherry tomatoes over the 15‐day storage period, see Figure 3B for details. The results indicate that both the control and treatment groups exhibited a trend of initially increasing respiratory intensity, followed by a subsequent decrease as storage time progressed. The peak respiratory intensity of the control group reached 3.81 mg CO₂/mg/gh on Day 6, whereas the treatment groups peaked on Day 9. Since cherry tomatoes are climacteric fruits, their respiratory and metabolic activities continue even after harvest, leading to the depletion of internal nutrients and accelerating fruit senescence. Therefore, to maintain the freshness of cherry tomatoes, it is essential to reduce their respiratory intensity. As shown in Figure 3C, the composite film treatment group with 0.5% flavonoid content exhibited the lowest peak respiratory intensity at 3.43 mg CO₂/mg/gh, which was significantly lower than that of the control group. This result demonstrates that the composite film effectively delayed the peak respiratory period of cherry tomatoes and inhibited their respiratory intensity.

#### 3.2.4 MDA

The MDA content in both the control and treatment groups exhibited an increasing trend with the extension of storage time, which can be seen in Figure 3D. However, starting from the 9th day, the MDA content in the control group was significantly higher than that in the treatment groups, indicating that the composite film effectively inhibited the increase of MDA in the fruit tissue, thereby contributing to the preservation of freshness. This effect is primarily due to the fact that, during storage, the cell membranes of fruits and vegetables are vulnerable to free radical attacks, leading to lipid peroxidation and the subsequent production of MDA. When the accumulation of MDA reaches a certain threshold, it is indicative of spoilage (Almajano et al. 2008). Therefore, MDA content can be used as a key indicator to assess the freshness of cherry tomatoes and evaluate the preservation efficacy of composite film. Notably, there were no significant differences in MDA content among treatment groups with varying flavonoid concentrations (*p* > 0.05). This observation suggests that changes in flavonoid content within the composite film did not markedly influence MDA levels in the cherry tomatoes, indicating that the presence of flavonoids may not play a critical role in reducing MDA accumulation during storage.

#### 3.2.5 Vc Content

Figure 3E shows in detail the changes in Vc content in cherry tomatoes over the 15‐day storage period. The Vc content in both the control and treatment groups exhibited an intial increase followed by a decline. This pattern can be attributed to the fact that Vc content typically peaks when cherry tomatoes reach full ripeness. As the storage period progresses, the maturity of the cherry tomatoes increases, leading to a corresponding rise in Vc content within the tissues. However, in the later stages of storage, Vc content begins to decline, due to the oxidation of Vc, which is triggered by the physiological activities of the fully ripened tomatoes (Al‐Dairi, Pathare, and Al‐Yahyai 2021). The control group reached its peak Vc content on Day 6, whereas the treatment groups peaked on Day 9, suggesting that the composite film effectively slowed the rapid increase in Vc content, thereby delaying the senescence of the fruits. At the same time, no significant differences were observed in Vc content across the five treatment groups with different flavonoid concentrations (*p* > 0.05). This result suggests that variations in the flavonoid content of the composite film did not significantly impact the Vc content in the fruit.

## 4 Conclusion

This study investigated the impact of PSF incorporation on the properties of PeCo composite films and their effectiveness in preserving cherry tomatoes. The findings revealed that the mechanical properties of the composite films deteriorated progressively with increasing flavonoid content. Among the various formulations, the PeCo‐0.1 composite film demonstrated the most favorable mechanical properties, with a tensile strength of 9.83 MPa and an elongation at break of 204.04%. However, the antioxidant capacity of the composite films improved with increasing flavonoid content. The PeCo‐0.5 composite film exhibited the highest DPPH free radical scavenging rate (21.37%) and superoxide anion free radical scavenging rate (15.08%). Fourier‐transform infrared (FTIR) spectroscopy and circular dichroism analyses revealed the specific protein structures within the composite film and the modifications induced by flavonoid interactions. In terms of cherry tomato preservation, the PeCo‐0.5 composite film demonstrated the most effective preservation performance. On Day 15, cherry tomatoes treated with the composite film exhibited the lowest PPO enzyme activity, weight loss rate (11.18%) and MDA content (13.33 μmol/g). Respiratory intensity analysis revealed that the composite film delayed the peak of respiration on Day 9, achieving the lowest peak value (3.43 mgCO_2_/mg/gh), PPO enzyme activity the bright color, smooth appearance, and fresh odor of the fruit throughout the 15‐day storage period. Given the excellent antioxidant properties and mechanical properties of the PeCo‐0.5 composite film in the preserving cherry tomatoes, its potential application in the preservation of other fruits and vegetables warrants further exploration. This could broaden its commercial application and meet the increasing market demand for high‐quality fruit and vegetable preservation. In the future, we will focus on enhancing the preservation performance of the composite film by incorporating other natural antioxidants or antibacterial agents. Additionally, we aim to gain insights into its versatility and effectiveness across different product types. Simultaneously, long‐term storage studies will be conducted to evaluate the film's performance over extended periods, providing valuable data for its practical application in commercial environments.

## Author Contributions

**Yuying Li:** data curation (equal), formal analysis (equal), investigation (equal), visualization (equal), writing – original draft (equal). **Kexin Jiang:** methodology (equal), software (equal), visualization (equal). **Qian Mao:** formal analysis (equal), funding acquisition (equal), resources (equal), writing – review and editing (equal). **Qi Zhao:** funding acquisition (equal), methodology (equal), supervision (equal), writing – review and editing (equal).

## Conflicts of Interest

The authors declare no conflicts of interest.

## Data Availability

Data will be made available on request.
